# Saudi mothers' preferences about breaking bad news concerning newborns: a structured verbal questionnaire

**DOI:** 10.1186/1472-6939-12-15

**Published:** 2011-08-23

**Authors:** Sameer Y Al-Abdi, Eman A Al-Ali, Matar H Daheer, Yaseen M Al-Saleh, Khalid H Al-Qurashi, Maryam A Al-Aamri

**Affiliations:** 1 Department of Pediatrics, King Abdulaziz Hospital, Al-Ahsa, Saudi Arabia; 2 Department of Nursing, King Abdulaziz Hospital, Al-Ahsa, Saudi Arabia

## Abstract

**Background:**

Breaking bad news (BBN) to parents whose newborn has a major disease is an ethical dilemma. In Saudi Arabia, BBN about newborns is performed according to the parental preferences that have been reported from non-Arabic/non-Islamic countries. Saudi mothers' preferences about BBN have not yet been studied. Therefore, we aimed to elicit the preferences of Saudi mothers about BBN concerning newborns.

**Methods:**

We selected a convenience sample of 402 Saudi mothers, aged 18-50 years, who had no previous experience with BBN. We selected them via a simple number-randomization scheme from the premises of a level III Saudi hospital between October of 2009 and January of 2011. We used a hypothetical situation (BBN about trisomy 21) to elicit their preferences about BBN concerning newborns via a structured verbal questionnaire composed of 12 multiple-choice questions. We expressed their preferences as percentages (95% confidence interval), and we used the Kendall's W test (W) to assess the degree of agreement in preferences.

**Results:**

The Saudi mothers preferred that BBN be conducted with both parents together (64% [60-69]), albeit with weak levels of agreement (W = 0.29). They showed moderate agreement in their preferences that BBN should be conducted early (79% [75-83], W = 0.48), in detail (81% [77-85], W = 0.52), in person (88% [85-91], W = 0.58), and in a quiet setting (86% [83-90], W = 0.53). With extremely weak agreement, they preferred to have a known person present for support during BBN (56% [51-61], W = 0.01), to have close bodily contact with their babies (66% [61-70], W = 0.10), and to have no another patients present (64% [59-68], W = 0.08). They showed moderate levels of agreement in their desires to detail, in advance, their preferences about process of BBN by giving a reversible, written informed consent that could be utilized for guidance, if needed (80% [76-84], W = 0.36).

**Conclusions:**

In our experience, Saudi mothers' preferences about BBN concerning newborns are varied, suggesting that a "one-size-fits-all" approach is inappropriate. A reversible, written informed consent detailing their preferences about BBN that would be kept in their medical records and utilized for guidance, if needed, may be the best solution, given this level of diversity. These findings merit further study.

## Background

Breaking bad news (BBN) to parents whose newborn has a major disease is an ethical dilemma with a diverse set of associated cultural beliefs [1-7]. This type of disclosure can be stressful for both health professionals and parents because it encompasses a mixture of happiness (giving birth) and great sadness (unexpected bad news). In Saudi Arabia, the practice of BBN to parents involves four significant factors. The first factor is the husband's guardianship over his wife and the parental authority that is practiced in most Arabic/Islamic countries, including Saudi Arabia. It is common in Saudi Arabia for a husband to ask a physician to disclose bad news to him first, after which he will decide how this news should presented to his wife [1]. The second factor is the reported personal experiences of the health-care professionals involved [1,2]. The third factor is the reported parental preferences about BBN from Western countries [3-7]. Most of these reports have surveyed parents about previous experiences with BBN; therefore, their findings may represent a mixture of information regarding the process of BBN and reports influenced by the prior receipt of bad news. It is unknown whether these preferences, originating from developed countries with diverse cultural contexts, apply to mothers from a conservative, Arabic/Islamic developing country. The fourth factor is the general, heuristic recommendations for BBN about serious diseases from the Saudi Commission for Health Specialists (SCHS) [8]. To help address this ethical dilemma more competently and effectively, therefore, we conducted this cross-sectional survey to elicit Saudi mothers' preferences about BBN concerning newborns and to assess the degree of agreement on these preferences.

## Methods

We conducted this cross-sectional interview at a level III Saudi hospital, King Abdulaziz Hospital for the National Guard (KAH), in the Al-Ahsa area, between October of 2009 and January of 2011. The participants were the mothers of children admitted to the pediatrics ward and mothers in the postnatal ward. We recruited a sample of convenience from this population, using a simple random-number generator to decrease the inherent selection bias of this sampling method. We used the following inclusion criteria: 1) Saudi; 2) aged 18-50 years; 3) no previous experience with BBN; 4) not a health care provider; and 5) able to give verbal consent.

This interview was performed using a structured verbal questionnaire administered in Arabic. We developed the study questionnaire based on current practice in Saudi Arabia and on the available literature [1-9]. The questionnaire consisted of 12 multiple-choice questions (Q) and 29 responses; each Q addressed one domain of BBN. We presented nine questions (Q1, Q3, Q4, Q6, and Q8-12) to all of the mothers; based on their responses, we then presented the remaining three questions (Q2, Q5, and Q7) to subgroups of them. The English translation of the questionnaire is shown in Table 1; a reverse translation into Arabic was conducted by an independent party. BBN was defined as the process of communicating the diagnosis of a disease that is potentially life-threatening or would significantly impair the newborn's development or well-being. We used BBN about a newborn with trisomy 21 (Down's syndrome) as a hypothetical situation for eliciting the personal preferences of the Saudi mothers about BBN. Breaking diagnosis of trisomy 21 itself and its features including mental retardation and congenital heart diseases were the hypothetical bad news. We conducted 6 mock interviews before commencing the study to ensure that the questionnaire was appropriate and understandable. This study was an Institutional Review Board (IRB) exempt as an interview and all mothers gave verbal consent. Those who declined participation were not asked to explain their refusal to participate in the study.

**Table 1:** The interview questionnaire and preferences of the Saudi mothers (n = 402) about breaking bad news (BBN) concerning their newborns

Interview Questionnaire	Preferences*	Kendall's W test
1. **The BBN process should initially involve whom?**		
A. Myself and my husband together in the same setting.	64% (60-69)	0.29 Weak agreement p < 0.001
B. My husband first, and he will decide about the process of BBN to me.	17% (13-21)	
C. Myself first.	16% (13-20)	
D. The most responsible physician should decide.	3% (2-5)	

2. **Would you agree if your husband requested to disclose the bad news to you through him in order to minimize your trauma?^¶^**		
A. Yes.	24% (20-29)	0.27 Weak agreement p < 0.001
B. No.	76% (72-81)	

3. **How best should the BBN be delivered?**		
A. In person.	88% (85-91)	0.58 Moderate agreement p < 0.001
B. In person or over the phone.	12% (9-16)	

4. **When would you prefer the BBN to be?**		
A. Early on, as soon as the bad news was identified.	79% (75-83)	0.48 Moderate agreement p < 0.001
B. The next day during routine postnatal rounds.	16% (12-20)	
C. The most responsible physician should decide.	5% (3-7)	

5. **By whom would you like to be informed if the most responsible physician is not on-site, such as outside regular working hours?^‡ ^**		
A. The most responsible physician, even if BBN needs to be postponed to the next day until he/she will be around.	50% (44-56)	0.14 Very weak agreement p < 0.001
B. Any physician who has a good understanding of the disease.	41% (35-47)	
C. The most responsible physician should decide.	9% (6-13)	

6. **How detailed do you prefer BBN to be?**		
A. Detailed.	81% (77-85)	0.52 Moderate agreement p < 0.001
B. Brief.	16% (13-20)	
C. The most responsible physician should decide.	3% (2-5)	

7. **How do you prefer the details to be communicated to you?^§^**		
A. All at once during the first meeting.	58% (52-63)	0.03 Very weak agreement p = 0.005
B. Gradually.	42% (37-48)	

8. **Do you prefer the BBN meeting to be quiet and free even from minor distractions, such as the informer answering non-urgent pager/phone calls?**		
A. Yes.	86% (83-90)	0.53 Moderate agreement p < 0.001
B. Neutral.	14% (11-18)	

9. **Do you prefer during BBN to have close bodily contact with your baby such as holding him/her in your lap?**		
A. Yes.	66% (61-70)	0.10 Very weak agreement p < 0.001
B. No.	34% (30-39)	

10. **Do you prefer during BBN that one of your relatives or friends be there as a support person?**		
A. Yes.	56% (51-61)	0.01 Very weak agreement p = 0.02
B. No.	44% (39-49)	

11. **Would you prefer during BBN that no another patient or his/her relatives be present?**		
A. Yes.	64% (59-68)	0.08 Very weak agreement p < 0.001
B. Neutral.	36% (32-41)	

12. **Would you agree to detail in advance your preferences regarding the process of BBN by giving a reversible, written informed consent which will be kept in your medical record and utilized as guidance if needed?**		
A. Yes.	80% (76-84)	0.36 Moderate agreement p < 0.001
B. No.	20% (16-24)	

*Expressed as a percentage (95% confidence interval).

^¶ ^Question 2 was presented only to those whose did not prefer response B in Question 1 (n = 335).

^‡ ^Question 5 was presented only to those who preferred BBN to be in person and early (Response A in both Questions 3 and 4) (n = 283).

**^§ ^**Question 7 was presented only to those who preferred BBN to be detailed (Response A of Question 6) (n = 325).

The preference rates were calculated by the number of mothers who chose a preference divided by all the participants who completed the questionnaire. We expressed the preferences as a percentage rounded to the nearest whole number and calculated an associated 95% confidence interval (95% CI). We used the T-test to compare mean age. We used the Kendall's W test (coefficient of concordance) to assess the degree of agreement on the preferences. Responses of each question were the dependent variables. We interpreted the Kendall's W test according to the following rule of thumb: ≤ 0.10 was extremely weak agreement, 0.11-0.30 was weak agreement, 0.31-0.50 was moderate agreement, 0.51- 0.70 was strong agreement, and 0.71-9.0 was extremely strong agreement [10]. We assessed the associations between the mothers' demographics (independent variables) and their preferences by using multivariate logistic-regression models to calculate odds ratios (ORs) and 95% CIs. We assessed the associations between demographics (independent variables) for each preference, and the significant interactions were included in the models. We used the likelihood ratio test to assess the significance and the Hosmer-Lemeshow test to assess the goodness-of-fit of the models. We considered a two-sided p-value < 0.05 to be statistically significant. All the statistical analyses were performed using SPSS 16 (SPSS, Inc., Chicago, IL).

## Results

We randomly selected 463 Saudi mothers to approach; of these, 402 (87%) were enrolled, and 61 (13%) were excluded from the study. The reasons for exclusion were as follows: refusal to participate (n = 48); previous experience with BBN (n = 12); and being a health-care provider (n = 1). Some of the mothers volunteered their reasons for refusing to participate in the study. These reasons included the following: they were discharged and on their way home (n = 11), they were tired after the delivery (n = 9), or they felt that the topic was too frightening (n = 8). Table 2 shows the demographic variables of the Saudi mothers who completed the study. All of the participants were urban from the Al-Ahsa area; 32.6% were originally Bedouin (nomadic) or from other areas of Saudi Arabia, however. The college-graduate mothers were younger than the non-college graduates (a mean of 29 [95% CI, 28-30] vs. a mean of 32 [95% CI, 31-33] years, p < 0.001).

**Table 2:** The demographic variables of the Saudi mothers (n = 402)


Mean age (95% confidence interval)	31 (30-32)
Total urban origin	305 (75.9%)
Originally from Al-Ahsa area	271
Originally from other areas	34

Total Bedouin origin	97 (24.1%)
Originally from Al-Ahsa area	48
Originally from other areas	49

Education level	
Illiterate	51 (12.7%)
Primary school	44 (11.0%)
Intermediate school	43 (10.7%)
High school	101 (25.1%)
College graduate	163 (40.5%)

Employed	71 (17.7%)
Less than college education	6
College graduate	65

Table 1 shows the mothers' preferences about BBN concerning newborns and the degrees of agreement in their preferences. The mothers preferred BBN to both parents together, although the level of agreement was weak. Only 37% of them accepted the practice of BBN to their husbands first either as their preference or as something they would agree to if it was their husbands' request. The mothers showed a moderate level of agreement in preferring that BBN be conducted early, in a detailed fashion, in person, and in a quiet setting. Those who preferred that BBN be conducted in person and as soon as the bad news was identified (n = 283) showed an extremely weak level of agreement on the preferred informer when the most responsible physician was not available. Of these, 50% modified their preference and accepted postponing the process of BBN to the next day. Among those who preferred that BBN be conducted in detail (n = 325), there was an extremely weak level of agreement on whether the details should be communicated all at once or gradually. The mothers preferred to have a known person for support during BBN, close bodily contact with their baby, and no another patients present, although the level of agreement was extremely weak. They showed a moderate level of agreement on choosing to determine, in advance, their preferences about BBN by giving reversible, written informed consent, to be kept in their medical record and utilized for guidance if needed.

### Demographics-preferences correlations

There was multicollinearity between the area of origin and both the urban origin and education level as well as between education level and employment status. Therefore, we only included the following three demographic variables in our logistic regression models: employment status (employed vs. unemployed), ethnicity (urban origin vs. Bedouin origin), and age as continuous variable. We did not observe any significant interactions between these demographic variables.

Table 3 shows results of multivariate logistic-regression models. The odds of preferring BBN to both parents together among the employed were 1.90 times those of the unemployed (p = 0.98 for the Hosmer-Lemeshow test), but the model had borderline significance (p = 0.053 for the likelihood ratio test). The odds of preferring BBN to the mother first among the employed were 0.21 times that of the unemployed (p < 0.001 for the likelihood ratio test; p = 0.64 for the Hosmer-Lemeshow test). The odds of preferring the presence of a known person for support during BBN among those with urban origins were 0.41 times that of those with Bedouin origins (p = 0.001 for the likelihood ratio test; p = 0.48 for the Hosmer-Lemeshow test). The odds of preferring having no another patients present during BBN among the employed were 1.94 times that of the unemployed and among those with urban origins were 1.78 times that of those with Bedouin origins (p = 0.04 for the likelihood ratio test; p = 0.42 for the Hosmer-Lemeshow test). The odds that a respondent was willing to give reversible, written informed consent in advance about BBN preferences among the employed were 0.45 times that of the unemployed (p = 0.02 for the likelihood ratio test; p = 0.65 for the Hosmer-Lemeshow test).

**Table 3:** Results of multivariate logistic-regression models

Preferences	Adjusted odds ratio (95% confidence interval)	p value
Breaking bad news (BBN) to both parents together		
Employed mothers	1.90 (1.05-3.43)	0.03
Urban mothers	1.33 (0.83-2.14)	0.32
Age	0.99 (0.47-1.20)	0.23

BBN to the husband first		
Employed mothers	0.67 (0.32-1.44)	0.31
Urban mothers	1.16 (0.62-2.18)	0.64
Age	0.98 (0.95-1.02)	0.39

BBN to the mother first		
Employed mothers	0.21 (0.06-0.68)	0.01
Urban mothers	0.57 (0.31-1.02)	0.06
Age	1.04 (1.00-1.07)	0.05

Would agree if their husbands' request to disclose the bad news to the mother through him		
Employed mothers	0.67 (0.37-1.19)	0.17
Urban mothers	1.49 (0.85-2.60)	0.16
Age	1.02 (0.98-1.05)	0.37

BBN in person		
Employed mothers	1.58 (0.64-3.89)	0.32
Urban mothers	0.91 (0.44-1.86)	0.79
Age	0.96 (0.95-1.03)	0.47

BBN as soon as possible		
Employed mothers	0.79 (0.43-1.45)	0.44
Urban mothers	1.10 (0.63-1.93)	0.74
Age	0.97 (0.94-1.00)	0.05

BBN by the Most responsible physician		
Employed mothers	1.44 (0.77-2.68)	0.26
Urban mothers	0.94 (0.54-1.61)	0.81
Age	1.01 (0.97-1.04)	0.69

BBN in detail		
Employed mothers	0.93 (0.49-1.79)	0.84
Urban mothers	1.33 (0.76-2.34)	0.31
Age	1.01 (0.98-1.05)	0.53

BBN all at once		
Employed mothers	0.70 (0.39-1.24)	0.22
Urban mothers	1.34 (0.67-1.92)	0.63
Age	0.99 (0.96-1.02)	0.51

BBN in quiet environment		
Employed mothers	0.51 (0.26-1.00)	0.05
Urban mothers	1.14 (0.59-2.21)	0.69
Age	1.01 (0.97-1.05)	0.59

Close bodily contact with your baby during BBN Employed mothers	0.77 (0.46-1.32)	0.35
Urban mothers	0.88 (0.54-1.43)	0.60
Age	1.02 (0.99-1.05)	0.22

Present of a known person for support during BBN		
Employed mothers	0.66 (0.39-1.12)	0.12
Urban mothers	0.41 (0.25-0.67)	< 0.001
Age	1.00 (0.97-1.03)	0.94

No another patient in same room during BBN		
Employed mothers	1.94 (1.08-3.51)	0.03
Urban mothers	1.78 (1.11-2.84)	0.02
Age	1.01 (0.98-1.03)	0.72

To give reversible, written informed consent		
Employed mothers	0.45 (0.25-0.80)	0.007
Urban mothers	1.70 (0.98-2.94)	0.06
Age	1.00 (0.97-1.04)	0.99

## Discussion

This study is the first to examine mothers' preferences about BBN concerning newborns in Saudi Arabia, a conservative Arabic/Islamic country. This study is also the first to assess the extent to which mothers agree on providing, in advance, their preferences about the process of BBN by giving reversible, written informed consent. This study demonstrated that Saudi mothers' preferences diverge on many aspects of BBN concerning newborns, suggesting that a "one-size-fits-all" approach is inappropriate. Their moderate level of agreement on using a reversible, written informed consent that can be utilized to guide the process of BBN, if needed, may indicate the best solution to this diversity in preferences.

In this study, we assessed only the mothers' preferences because of the husband's guardianship over his wife and the parental authority that is practiced in Saudi Arabia. The Saudi mothers demonstrated an extremely weak degree of agreement on 5 of the 12 BBN domains included in this study. These domains were the presence of a known person for support during BBN, close bodily contact with the newborn during BBN, the presence of another patient during BBN, the identity of the informer when the most responsible physician is not available, and the practice of communicating the details gradually or all at once. Our study suggests that the current common practice in Saudi Arabia of BBN to the husband first needs to be replaced by BBN to both parents together. Only 37% of the mothers preferred BBN to their husbands first either as their primary preference or as something they would accept at their husbands' request, supporting the need for this change. It has been reported that BBN is less stressful when it is conducted with both parents together [5]. This finding may explain why the employed mothers, who were already under stress from their employment, showed greater preference for BBN to both parents together and less preference for BBN to the mothers first, as compared to the unemployed mothers. Because the essential family script is nuclear among the urban population and extended among Bedouins, it was not surprising that the urban Saudi mothers showed less of a preference than did the Bedouin mothers for having a known person for support during BBN. The demographics of Saudi women are rapidly changing towards more urban status, college-education, and employment outside the home. Therefore, our associations between these demographics and the mothers' preferences concerning BBN suggest that a continued drift in these preferences will be observed as these demographic changes.

The Saudi mothers showed preferences similar to those of their counterparts in Western countries; they preferred that BBN be conducted early, in detail, in person, and in a quiet setting [1-7]. By contrast, they showed less of a preference than did their Western counterparts for BBN to both parents together and for gradual communication. Husband's guardianship over his wife and the parental authority that is practiced in Saudi Arabia may contribute to these differences [1]. They also showed less of a preference than did their Western counterparts for having a person present for support and for close bodily contact with their babies during BBN [6,7]. We have no clear explanation for these differences as we did not tailor our survay to rationalize the preferences. However, the diverse cultural and religious beliefs towards BBN likely contributed to these differences [1-7]. Saudi parents usually keep bad news related to their children secret even from the intimate family members and friends [1]. Muslims believe in God "Allah", destiny "Qadar" and divine decree "Qada" as well as they view diseases as expiation for sins. All these beliefs provide them with internal contentment [1,2].

Some of the mothers' preferences are in disagreement with the general recommendations about BBN provided by the SCHS [8]. The SCHS recommends that the most responsible physician decide on the timing of BBN, the kind of information that should be revealed, and the involvement of relatives. However, the mothers' responses in Q1 and Q4-Q6 showed that these approaches were the least preferred options. The SCHS suggests that BBN should proceed gradually, but this approach was preferred by less than one half of the mothers. It suggests that BBN should be brief, a recommendation that also conflicts with our finding that a majority of the mothers preferred BBN to be detailed. The SCHS assumes that the most responsible (treating) physician is the one responsible for the process of BBN. We designed Q5 to determine which factor is more important for the mothers, the physician responsible for or the timing of BBN. Unfortunately, due to the weak level of agreement in their responses, we failed to thoroughly address this issue. The general nature of the recommendations of the SCHS may explain the degree of disagreement between these recommendations and the mothers' preferences about BBN concerning newborns.

The above-mentioned diversity in preferences may appear to be a barrier to BBN satisfactorily and efficiently. Using a reversible, written informed consent document, to be kept in the medical record and utilized as guidance if needed, may be the most effective tool for overcoming this obstacle. Such a document would allow the mothers to designate their preferences in advance. It has been suggested that informed consent about BBN may be a logically and ethically sound process for competent patients, and we agree with the idea that such informed consent merits further study [9]. Admittedly, we have no explanation for why the employed mothers, who were mostly college graduates, were less willing to give such consent. This finding seems to contradict another study from Iran, which demonstrated that professionally employed mothers have significantly higher self-esteem and self-efficacy than their unemployed counterparts [11].

In addition to the inherent limitations of this type of study, we used a sample of convenience from one hospital, a technique which may have limited the generalizability of our findings. To help overcome this limitation, we selected our participants via a simple number-randomization scheme to reduce the selection bias from the sample. Fortunately, the demographics of our sample were similar to the overall population of Saudi mothers [12,13]. Additionally, our sample size was fairly large, given that 385 participants would be needed using random sampling to achieve a 5% margin of error at a 95% confidence level with 1 design effect and a 50% anticipated response. We also did not include all possible BBN domains, such as the presence of a social worker during BBN or the particular language or level of emotion used by the informer; these omissions may represent additional limitations of our study. We limited the number of survey items because we did not want the participants to be overwhelmed by a lengthy survey that might have decreased their overall compliance with the study.

## Conclusions

In our experience, Saudi mothers' preferences about BBN concerning newborns are varied, suggesting that a "one-size-fits-all" approach is inappropriate. The moderate level of agreement on using a reversible, written informed consent that can be utilized to guide the process of BBN, if needed, may indicate the best solution to this diversity in preferences. Although more research is needed in this important area, we hope that our findings will stimulate future reach and help health care providers in Saudi Arabia and similar communities to establish guidelines for effectively communicating bad news concerning newborns.

## Competing interests

The authors declare that they have no competing interests.

## Authors' contributions

SA designed the study, and all of the authors participated in the development of the survey and the administration of the interviews. SA entered and performed the statistical analysis of the data, and MA reviewed the data entry. The draft of the manuscript was written mainly by SA, and the other authors read, amended, and approved the final manuscript.

## Pre-publication history

The pre-publication history for this paper can be accessed here:

http://www.biomedcentral.com/1472-6939/12/15/prepub
